# Effect of the Dislocation Substructure Parameters of Hadfield Steel on Its Strain Hardening

**DOI:** 10.3390/ma16041717

**Published:** 2023-02-18

**Authors:** Alyona Russakova, Almira Zhilkashinova, Darya Alontseva, Madi Abilev, Alexandr Khozhanov, Assel Zhilkashinova

**Affiliations:** 1 National Scientific Laboratory for Collective Use, Sarsen Amanzholov East Kazakhstan University, 34 Tridtsatoy Gvardeiskoy Divizii Str., Ust-Kamenogorsk 070002, Kazakhstan; 2 School of Information Technologies and Intelligent Systems, D. Serikbayev East-Kazakhstan Technical University, 69 Protozanov Str., Ust-Kamenogorsk 070004, Kazakhstan; 3 Department of Analytical, Colloid Chemistry and Technology of Rare Elements, al-Farabi Kazakh National University, 71 al-Farabi ave., Almaty 050040, Kazakhstan

**Keywords:** Hadfield steel, strain hardening, dislocation substructure, transmission electron microscopy, twinning, strain-hardening coefficient

## Abstract

This article presents a study of changes in the microstructure of Hadfield steel depending on the tensile deformation and cold rolling with the strain/stress level. It has been established that the change in the “σ-ε” curve (at ε = 5%) is accompanied by a 1.5-times decrease in the strain-hardening coefficient. At ε = 0 to 5%, the structure contains dislocation loops, the interweaving of elongated dislocations, single-layer stacking faults. At ε = 5%, the structure contains multilayer stacking faults and mechanical microtwins. At ε > 5%, there is an intense microtwinning with no long dislocations and stacking faults. The most intense twinning develops in the range of deformation degrees of 5–20%, while the number of twins in the pack increases from 3–4 at ε = 10% to 6–8 at ε = 20%. When mechanical twinning is included, a cellular dislocation substructure begins to develop intensively. The cell size decreases from 700 nm at ε = 5% to 150 nm at ε = 40%. Twinning develops predominantly in systems with the largest Schmid factor and facilitates the dislocation glide. The results may be of interest to the researchers of the deformation processes of austenitic alloys.

## 1. Introduction

The austenitic high-manganese Hadfield steel (1.2% carbon and 12% manganese) occupies a special place among wear-resistant high-carbon manganese alloys. Due to high toughness, good flexibility, and wear resistance, Hadfield steel is widely used in various industrial applications (rock crushing and railroad crossing parts, excavator buckets, track tracks, etc.) [1,2,3]. In the casting of Hadfield steel without heat treatment, the structure of steel consists of austenitic and carbide phases. The carbide phase usually precipitates along the grain boundaries and leads to the brittleness of the casting. To eliminate this disadvantage, annealing at 1050–1150 °C (depending on the specific elemental composition of the steel) with water quenching is usually used [3]. The structure of Hadfield steel should be austenitic single-phase to ensure optimal impact strength [4,5,6]. A small amount of fine carbides in an austenitic matrix with evenly distributed carbon and manganese is acceptable. On the other hand, the presence of carbides increases the abrasion resistance [7,8,9]. Thus, Hadfield steel combines abrasive wear resistance and high toughness.

A distinctive feature of Hadfield steel is its pronounced ability to strain harden. The main strengthening mechanism of Hadfield during plastic deformation is the pile-up dislocations (dislocation arrangements may develop highly compact tangles with an increase in the degree of deformation) and the formation of twins [10]. The crystallographic glide planes with a high dislocation density are generally called as slip bands [11]. In this case, the high rate of strain hardening of this steel is due to the deformation transformation of γ austenite into α- or ε-martensite, mechanical twinning, dynamic strain aging, and blocking of dislocations by stacking faults. The gliding dislocations are blocked at stacking faults [2,6,12]. Stacking fault energy (SFE) values can be controlled by certain methods within a certain range, since they are related to the chemical composition and mainly depend on the volume percentage of manganese and carbon [6,7,9,13]. It is also noted that the addition of nitrogen is promising for this steel, since it improves its mechanical strength without changing the ductility [12].

In [14,15], an approach was used based on comparing the dependence of strain hardening on the microstructure of the deformed material, while the total hardening of steel was considered as a linear superposition of the contributions of solid solution hardening, dislocations, and twins. It is noted that most of the contributions to hardening are associated with the action of a certain deformation mechanism, which corresponds to its own type of defective substructure, while the condition for the isolation of any stage of plastic flow is the constancy of the strain-hardening coefficient. Thus, it can be assumed that the stages of the deformation curve during plastic deformation of the Hadfield steel also depend on the type of dislocation structure, which determines the values of the strain-hardening coefficient in austenitic steels.

In the study of the dislocation structure of polycrystals of austenitic stainless steels, it was previously established that stacking faults are observed simultaneously with planar pile-up dislocations [14,15,16,17], and the role of stacking faults as strengthening elements of the dislocation structure was shown. If during the development of the dislocation structure upon deformation, microtwins are formed, they can lead to additional strengthening. In addition, an increase in the degree of plastic deformation in austenitic steels is accompanied by an increase in the stress level and a change in the deformation mechanism from slip to twinning, which can become the main deformation mechanism. On the other hand, an increase in the number of active twinning systems can determine the stages of the flow curves and strain-hardening coefficient. If twinning develops after slip deformation, there will be competition between cross slip and twinning [14,16,17]. The development of twinning in one system after slip will suppress the processes of cross slip and lead to an increase in the plasticity of crystals, that is, the TWIP (twinning induced plasticity) effect. A challenge for the structural application of TWIP steels is their low yield strength (~300 MPa) [18], arising from their single-phase microstructure, where the main strengthening mechanism is dislocation strengthening during the initial deformation stages [11], leading to increased yield strength and decreased ductility.

Bracke et al. [19] showed that at the degree of deformation ε = 0.10 twins appear in the form of straight lines. At this stage, direct deformation twins are already present in more than half of the grains. Only one twinning system is activated in each grain; the twinning system has a suitable orientation with respect to the rolling direction. Twins are formed on the {111} planes with the maximum obtained stress. In grains free of deformation twins, dense planar pile-up dislocations are observed.

Nikulina et al. [20] showed that the hardening of steel is due to the formation of dislocation tangles, generation of stacking faults, and twins. The steel hardening was caused by the formation of the pile-up dislocations and the generation of stacking faults and twins. It was found that regions with nanocrystalline structures with face-centered cubic (FCC) and hexagonal close-packed lattices were formed in Hadfield steel (13.42% Mn, 1.2% C, 0.71% Si, and Fe as the remainder) after 40% rolling deformation.

To increase the ductility of TWIP steel, Zhi et al. [21] introduced gradient substructures (GS) in Fe-22Mn-0.6C TWIP steel, and investigated the evolution and mechanical properties of GS. The uniaxial tensile tests showed that GS simultaneously obtained a combination of high yield strength and high tensile strength due to dislocation strengthening with the accumulation of high dislocation density and high ductility originated from gradient substructure-induced back stress hardening.

Kim et al. [11] used in-situ TEM for direct observation of dislocation plasticity in high-Mn lightweight steel and established that the plastic deformation of the austenitic Fe-Mn-Al-C steel was accommodated by the pronounced planar dislocation glide followed by the formation of slip bands and highly dense dislocation walls. During the plastic deformation of this steel, neither martensitic transformations nor deformation twins were observed, but the localized cross-slip of dislocations at the slip band intersection was observed, i.e., the slip bands propagated in the grain without blocking each other. Thus, it was confirmed that the enhanced strain-hardening behavior was attributed to the pronounced planar glide, which extended the deformation stage II region.

In the previous study [22], the patterns of changes in the microstructure and phase composition of high-manganese steel depending on the content of the main components (Mn, C) were established. An increase in C content in steel stabilizes austenite, thus, steels close in composition to Hadfield steel with Mn content from 10 wt.% to 18% and from 1.0–1.2 wt.% C, remain austenitic not only after hardening, but also after plastic deformation by rolling with a degree of deformation up to 60%. In this case, the value of the average scalar density of dislocations at equal degrees of rolling deformation was directly proportional to the concentration of C, while an increase in the Mn content had practically no effect on the average scalar density of γ-phase dislocations.

At present, the role of dislocation and twin substructures in the strain-hardening behavior is generally well modeled and studied, a complete analysis of the experimental data and a generalization of modern concepts in the field of deformation behavior of face-centered cubic alloys of g-Fe with a high manganese content was performed by De Cooman et al. [10]. There are significant differences between the models proposed for the evolution of the microstructure during the deformation of TWIP steels and the concomitant behavior of strain hardening.

The role of dislocations is an important parameter to be studied not only for Hadfield steel but for other alloys too. For example, the role of dislocations and related boundaries on the hydrogen embrittlement behavior of a cold-rolled 7xxx series Al alloy was studied in [23]. A cold-rolled 7xxx series aluminum alloy contains hydrogen at the statistically stored dislocations, high-angle boundaries, and interstitial lattice. Most of the hydrogen atoms that are trapped at dislocations are found in statistically stored dislocations. With the further cold rolling reduction, the normalized hydrogen content determined by statistically stored dislocation density remained unchanged. Therefore, statistically stored dislocations are not the primary trap site influencing the alloy’s hydrogen embrittlement behavior.

However, experimental characteristics of the evolution of the deformation substructure, as well as a detailed quantification of their respective roles in the strain hardening of Hadfield steel during tensile deformation, are still rarely reported in the literature.

Therefore, this study aims to establish the structural changes in Hadfield steel as a result of mechanical stress by tension and rolling, to evaluate the quantitative parameters of its dislocation substructures, and to evaluate the influence of the deformation mechanism (sliding and twinning) and the substructures formed during deformation on the strain hardening of this steel. The experimental facts and theoretical considerations of this article contribute to the current picture of deformation processes in TWIP steels, in which deformation twinning plays an important role.

## 2. Materials and Methods

Austenitic Hadfield steel (110G13L grade, analogue of BW10 grade) was used as the study material. The elemental composition of steel is given in Table 1. All samples were purchased from VostokMashZavod JSC (Ust-Kamenogorsk, Kazakhstan). The chemical composition of the samples was provided by the manufacturer and checked by the energy dispersive X-ray analysis (EDX) using a scanning electron microscope JSM-6390LV (JEOL, Tokyo, Japan) with an energy dispersion analysis add-on unit (Inca energy, Oxford Instruments, Abingdon, UK).

All ingots were homogenized and normalized. Thermal heating of steel samples (16 × 22 × 0.5 mm^3^) was carried out at a temperature of 1050 °C for 30 min in a laboratory tubular electric furnace SUOL-0.4.4/12-M2-U4.2 (Tehnoterm, Istra, Russia) in a vacuum. The temperature in the furnace was measured and controlled by a precision temperature controller VRT-2 using two thermocouples of the TPP 1378 type (Tekhnogazkomplekt, Kharkov, Ukraine). The accuracy of temperature control and maintenance was ±0.5 °C, and the measurement error at the temperature of the upper measurement limit of 1300 °C was no more than ±3 °C, i.e., the total temperature measurement error in this furnace did not exceed ±4 °C. The state of the samples was fixed by quenching them in water at room temperature.

Cold deformation by rolling of hardened samples was carried out at room temperature on a manual laboratory mill to a sample thickness of 0.5 mm. To obtain the required degree of deformation, the rods of the studied alloys were cut into bars of different thicknesses before rolling.

Mechanical tests were also carried out at room temperature on the samples with dimensions of 0.5 × 15 × 75 mm^3^. The tests consisted of uniaxial static tension of the samples to rupture on a UPR-60 mechanical testing machine (Polyani type) with measurement of the yield stress σ (MPa). The stress exceeding the yield stress was determined by the ratio of the load to the actual cross-sectional area of the sample with uniform deformation at a rate of 0.2·10^−3^ s^−1^. The tension axis was oriented along the rolling direction. Based on the results of the experiments, graphs of the dependences of the flow stress σ (MPa) on the degree of plastic deformation ε (%) were plotted and the strain hardening coefficients Θ (Θ = dσ/dε) for the Hadfield steel were calculated.

For TEM analysis, the samples were prepared by mechanical grinding and electrolytic thinning in a mixture of hydrogen peroxide in phosphoric acid at a temperature of 80 °C and twin jet electropolishing. The electropolishing agent was a supersaturated solution of chromic anhydride in phosphoric acid at a temperature of 60 °C and with an electric current density of 0.5–0.7 A/cm^2^. Sample foils were examined for narrow twins and dislocation structures in a JEM-2100 (JEOL, Japan) analytical transmission electron microscope operated at 200 kV. JEM-2100 was equipped with a STEM system and an energy-dispersive micro(nano) system Inca Energy TEM 350 Analysis (Oxford Instruments, UK). The method of arbitrary secant lines was used to determine the volume ratio of dislocation substructure and twins by TEM images. At least five TEM images for each type of sample with the minimum of 15 arbitrary secant lines were analyzed.

Samples for optic microscopy using Neophot-21 (Carl Zeiss, Jena, Germany) were prepared by mechanical and electrolytic polishing and etching as detailed in [22].

The volume fractions of deformation microtwins were determined by the planimetric method [24]. To determine the volume fraction (P_v_) of the dislocation substructure (DSS) formed during deformation, we used the planimetric method described in [25,26,27] for determining the volume fraction from random sections, based on measuring the fraction of the foil area occupied by a certain type of DSS. The scalar dislocation density was measured by the secant method with a correction for the invisibility of dislocations, as described in [28].

To study the DSS evolution with the strain/stress level, we measured such parameters as the density of dislocations [24,25] and twin area fraction [29]. The excess dislocation density ρ± = ρ+ − ρ− (ρ+ and ρ− are the density of positively and negatively charged dislocations, respectively) was measured locally along the misorientation gradient and was calculated using the Equation (1):

(1) $$\rho_{\pm }=\frac{1}{b}\cdot \frac{\partial \varphi }{\partial l},$$

where *b* is the Burgers vector of dislocations; ∂*φ*/∂*l* is the gradient of the curvature of the foil or the curvature-torsion of the crystal lattice *χ*. The value *χ* = ∂*φ*/∂*l* was determined by shifting the extinction contour (Δ*l*) at a controlled angle of inclination of the foil (Δ*φ*) in the microscope column using a goniometer.

## 3. Results and Discussion

**Figure 1:**
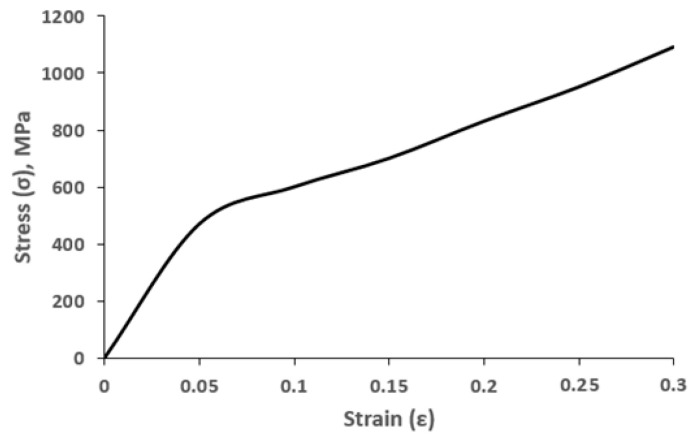
Tensile stress-strain (σ − ε) curve for Hadfield steel, deformed by tension at 0.2 × 10^−3^ s^−1^.

The results of tensile tests of Hadfield steel samples are presented by the tensile stress-strain (σ − ε) curve as shown in Figure 1.

Upon deformation of Hadfield steel at the constant tensile rate of 0.2·10^−3^ s^−1^, there are two stages of plastic flow development II_1_ and II_2_ with different strain hardening coefficients (Θ(II_1_) = 3·10^3^ MPa and Θ(II_2_)= 2.1·10^3^ MPa, respectively). The inflection on the σ − ε curve corresponds to the value of the degree of tensile deformation ε = 0.05 (5%) and, presumably, corresponds to the moment of inclusion in the twinning deformation. The shape of the curve in Figure 1 is in good agreement with the true stress-strain curve of the high-Mn steel (Fe-32Mn-8.9Al-0.78 C (wt.%)) studied by in-situ TEM [11]. Authors noted that the ‘hump’ in the strain hardening rate curve implied a dominant hardening mechanism was activated with ongoing strain and provided evidence of the glide plane softening which extends the deformation stage II region.

**Figure 2:**
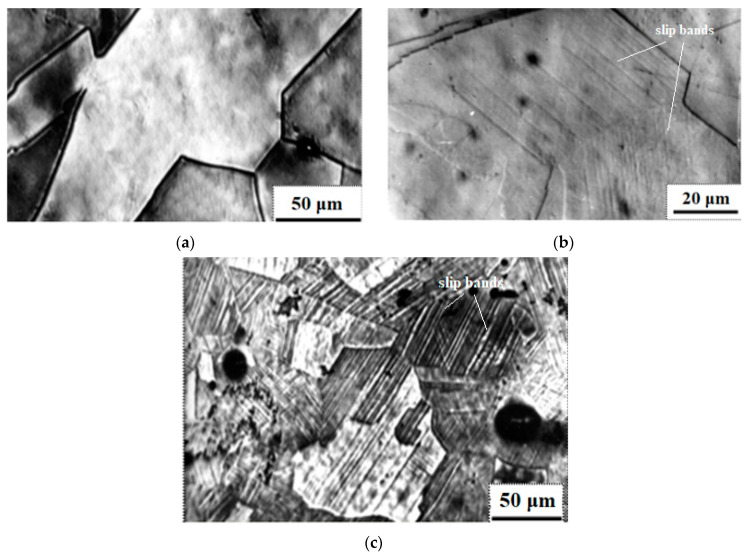
Images of the microstructure of steel after hardening (**a**), tensile 5% (**b**) and 20% (**c**) deformation.

Before deformation, the steel microstructure is represented by grains with an average grain size of 150 μm without carbides at the boundaries and without slip bands inside the grains (Figure 2a). Upon reaching the degree of tensile deformation of 5%, slip bands appear inside individual grains (Figure 2b), and upon tensile deformation of 20%, the number of slip bands increases, being observed in all grains, but the grains are still not elongated along the direction of deformation (Figure 2c).

**Figure 3:**
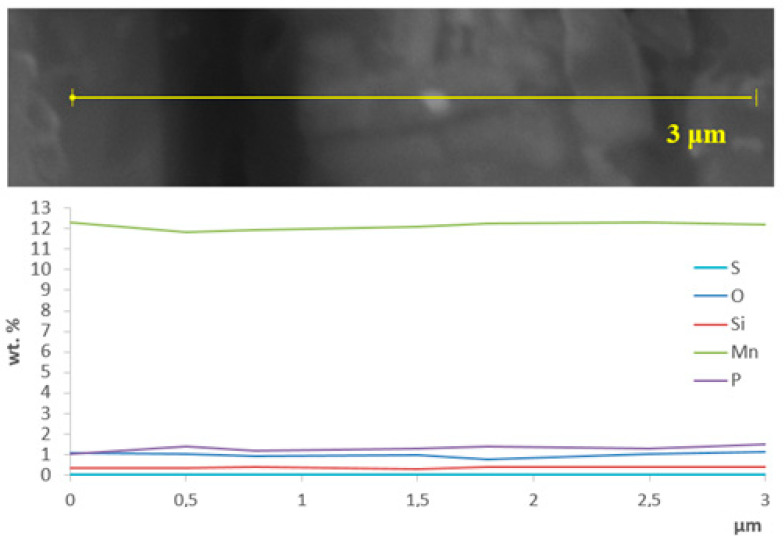
STEM image with EDX analysis of the distribution of elements for a sample of Hadfield steel.

The steel structure before deformation is homogeneous austenitic and the results of the EDX analysis of the distribution of elements in the selected direction (Figure 3) do not show any noticeable deviations from the steel composition indicated in Table 1. This confirms the homogeneity of the structure and absence of carbides at the boundaries observed by optic microscopy (Figure 2a).

**Figure 4:**
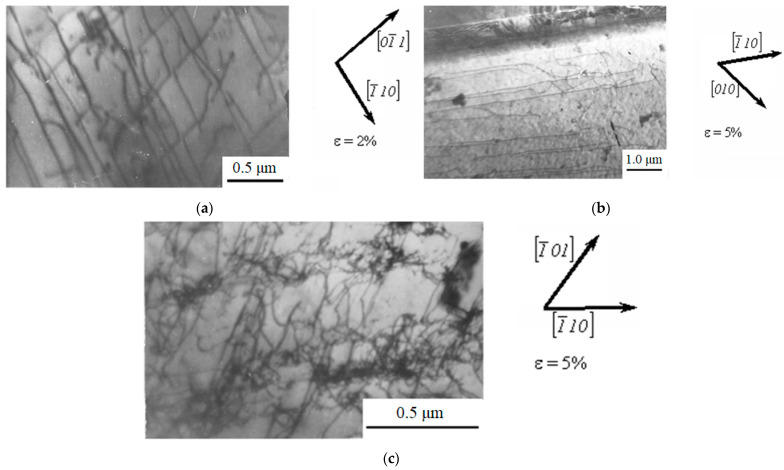
TEM images of the dislocation substructure of Hadfield steel deformed by tension with ε ≤ 5%: (**a**) long dislocations; (**b**) stacking faults, (**c**) tangles of dislocations.

TEM results showed that at the initial degrees of deformation (up to ε = 5%), the substructure of the material is represented by randomly distributed dislocations and individual mechanical microtwins (Figure 4).

The presence of regions with a predominance of long dislocations elongated along one or two <110> directions is typical for tensile deformation with ε < 5% (Figure 4a). These dislocations accumulate and form tangles, which are still weakly expressed at the initial degrees of plastic deformation. With an increase in the degree of deformation to 5%, single-layer stacking faults (Figure 4b) and tangles of dislocations (Figure 4c) appear in the structure.

**Figure 5:**
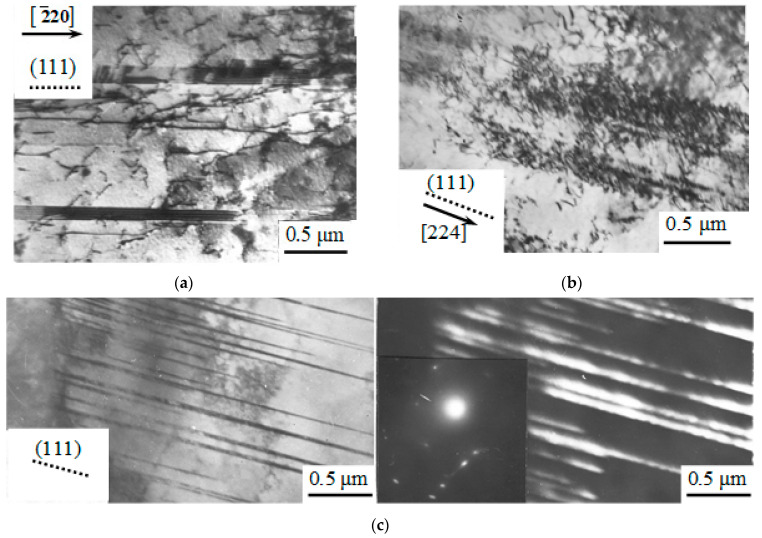
Stages of deformation twins formation in Hadfield steel deformed by tension with ε = 5% (**a**); ε = 8% (**b**); ε = 14% (**c**): single-layer (**a**), multilayer (**b**) stacking faults (incomplete twinning) and microtwins (**c**) (complete twinning). The dotted line marks the trace of the habit (111) plane.

Upon reaching ε = 5%, multilayer stacking faults and mechanical microtwins appear in the structure (Figure 5a). At tensile deformation of ε = 8%, TEM images show single-layer and multilayer stacking faults (Figure 5b) and completed microtwins at ε = 14% (Figure 5c). At ε > 5%, when microtwinning begins to develop intensively, there are no long dislocations and stacking faults in the material.

Thus, at the initial tensile deformation degrees within the initial grains, the formation of a dislocation substructure begins. With an increase in the degree of plastic deformation, the density of dislocations at the subboundaries increases, and the crystallographic misorientations between subgrains increase, which leads to a sharp change in the hardening coefficient when the degree of deformation reaches 5%, as shown in Figure 1.

In previous studies [12,30,31], the authors associated the linear nature of the hardening of Hadfield steel during deformation with sliding deformation and mechanical twinning, explaining that the hardening caused by a change in the microstructure at room temperature is explained by twinning in materials of this type in the same way as it is described for the Hall-Petch effect. The dynamic Hall-Petch effect from the microstructural refinement by the formation of the multiple domain walls is stated in [32], however, the long-range interaction between the pile-up dislocations is highlighted in the mechanisms in [11,33,34]. In contrast to [11], a sharp decrease in the strain hardening coefficient is observed in this experiment; therefore, the deformation mechanism requires more detailed consideration. The dependence, presented in Figure 1, is more likely to agree with the assumption of the occurrence of the TWIP effect in Hadfield steel, as described in [13,15,16,35,36].

**Figure 6:**
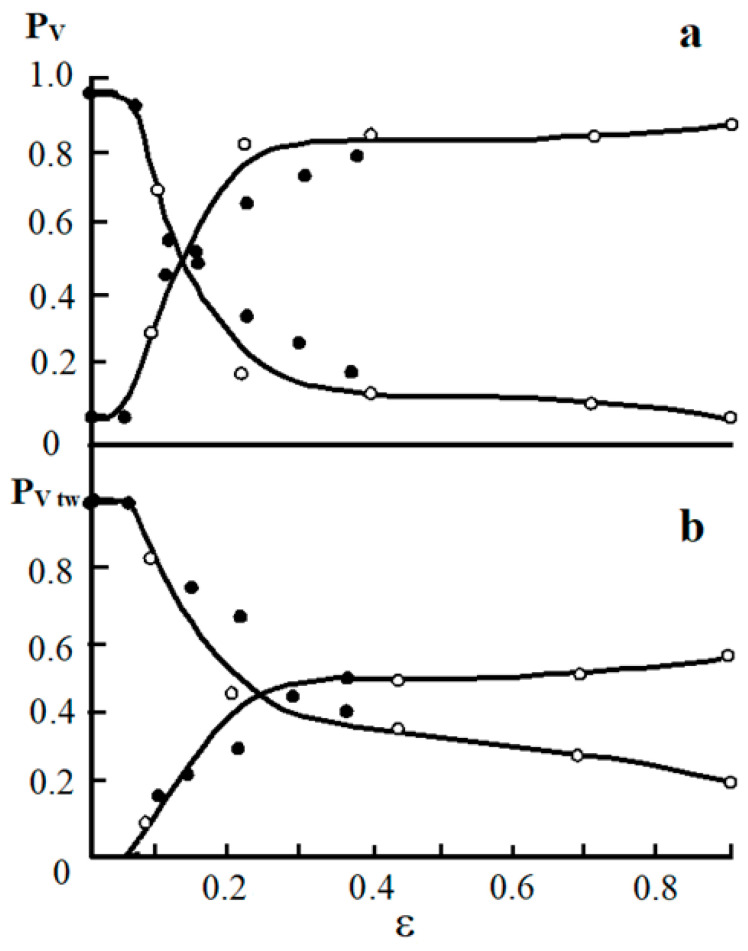
Twin area fraction of material covered by sliding (**a**) and twinning (**b**) depending on the degree of deformation: •—tensile deformation, ⸰—rolling deformation.

Figure 6 shows the dependences of the experimentally determined twin area fractions of the material covered by sliding (P_v_) and twinning (P_vtw_) (Figure 6a), as well as the dependence of the number of twins developing in systems 1, 2, and 3 (Figure 6b) on the degree of deformation both by tension and rolling.

**Figure 7:**
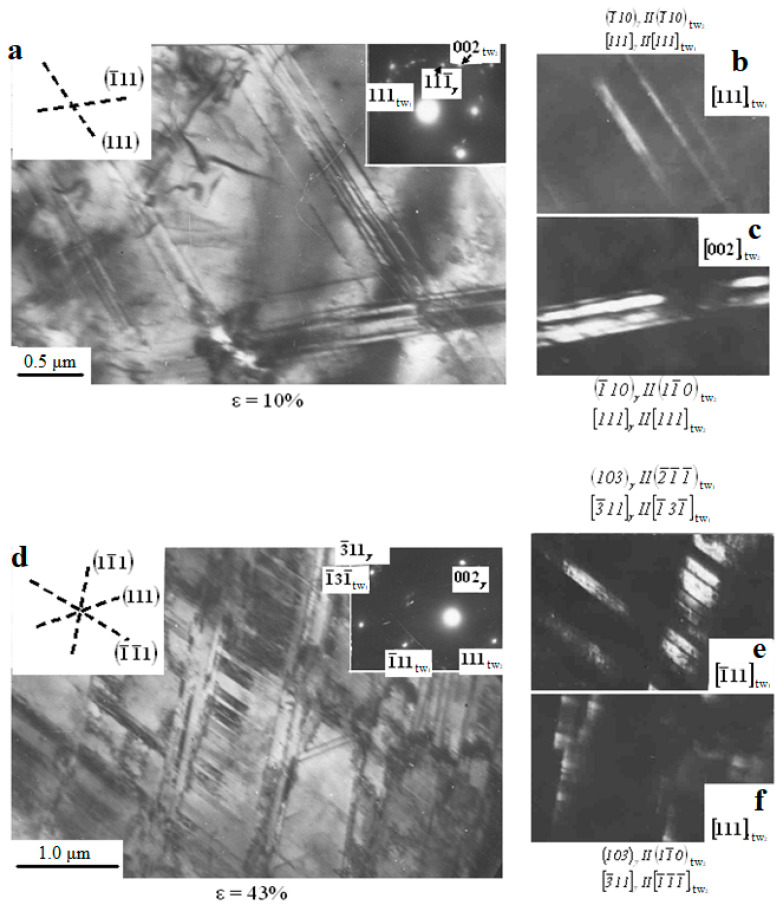
TEM micrographs of intersecting systems of microtwins in Hadfield steel after deformation (ε = 10%): the bright field—with electron diffraction pattern, where the arrows show the reflections of the γ-matrix and twins (**a**); the dark field is obtained in the reflections of twins [111] (**b**) and [002] (**c**); after deformation with ε = 43%, the bright field—with electron diffraction pattern (**d**), the dark field is obtained in the reflections of twins [$\bar{1}11$] (**e**) and [111] (**f**).

The results shown in Figure 6a confirm the above assumption that the moment when the twinning mechanism is included in the deformation (ε = 5%) coincides with the inflection on the σ − ε curve (Figure 1), corresponding to a decrease in the strain-hardening coefficient Θ by about 1.5 times. With an increase in the degree of plastic deformation, the fraction of the material engulfed in twinning increases. Twinning develops most intensively in the range of deformation degrees from 5% to 20%. One grain may contain mutually intersecting systems of microtwins (Figure 7).

**Figure 8:**
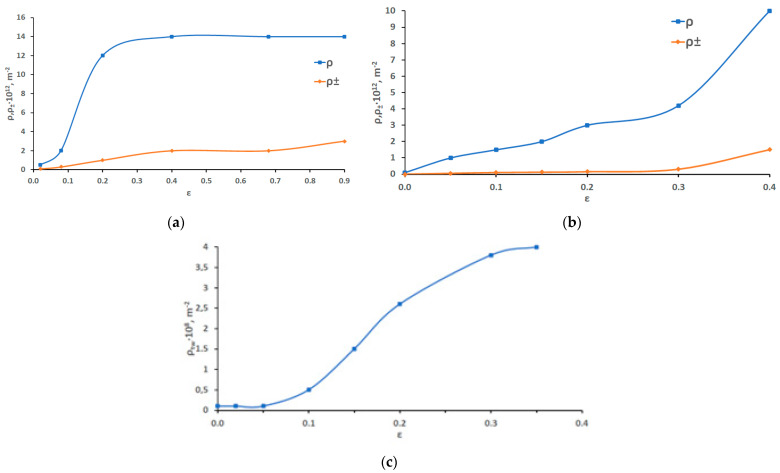
Dependences of scalar ρ and excess ρ_±_ dislocation density at rolling deformation (**a**), at tensile deformation (**b**) and ρ_tw_ (**c**) on the plastic deformation degree in Hadfield steel.

As seen in Figure 7, twins form packets. The formed twins acquire warped shapes when they are crossed by other twins and slip bands. The average number of twins per packet increases from 3–4 at ε = 10% (Figure 7a–c) to 6–8 at ε ≥ 20% (Figure 7d–f). The fraction of the material covered by two or even three twin systems increases with the degree of deformation, and the fraction of twins developing in one system decreases. For example, at the deformation degree of 20%, for samples deformed by rolling, the twin area fraction in three systems is about 2%; for two systems—about 10%, and for one system—about 80%. Then, when the degree of deformation reaches 40%, the area fraction of twins is 10% for three systems, 50%—for two, and 30%—for one system. 

Thus, a correlation is observed between changes in the substructure, the activation of new deformation mechanisms (namely, twinning), and stages of plastic flow. With an increase in the deformation degree, the development of twinning in several sliding systems and the formation of packets of microtwins is observed. The number of twins in the packets increases with increasing deformation, that is, the TWIP effect is presumably observed, accompanied by a decrease in the hardening coefficient by about 1.5 times.

In order to reveal the relationship between the stages of deformation and the quantitative parameters of the dislocation substructure, the following parameters were measured: scalar and excess density of dislocations and the density of tensile deformation twins. The dependence of the quantitative parameters of the substructure (scalar and excess density of dislocations and the density of deformation microtwins) on the plastic deformation degree is shown in Figure 8.

Figure 8 shows that a change in the method of deformation (tension, rolling) significantly affects the shape of the scalar dislocation density curve (Figure 8a). The dependence of the scalar dislocation density on the degree of tensile deformation is parabolic in nature, reaching a maximum value of the order of 10^13^ m^−2^, at the deformation with a degree of 40%, at which the destruction of most samples occurs under tension. Under tension, the parabolic growth of the scalar dislocation density ρ continues up to ε = 30%. At higher degrees of deformation, the accumulation of dislocations saturates, corresponding to values of 1.4·10^13^ m^−2^. Under tension, the destruction of the sample occurs before saturation of the dislocation density can be reached, and the growth rate of the scalar dislocation density is much lower than during rolling. The excess dislocation density grows along a similar curve with an increase in the degree of deformation by tension and rolling (Figure 8a). The obtained results are consistent with both the results of our previous studies [22], according to which, at ε = 40%, the scalar dislocation density in the Hadfield steel was about 1.2 × 10^13^ m^−2^, and with [36], where the dislocation density in the austenite before the martensitic transformation was found of the order of 10^13^ m^−2^ at room temperature. However, it can be assumed that by increasing the accuracy of measurements of the scalar dislocation density in coils, it would be possible to obtain higher values of ρ. Based on the methods for dislocation density estimation in highly compacted tangles proposed by Monteiro et al. [37], a dislocation density value of (4 ± 2)·10^15^ m^−2^ was preliminarily found to be the maximum limit for metal materials and it was indicated that iron–carbon martensite, which is the hardest known metal phase, has a dislocation density of the order of 10^15^ m^−2^. Thus, the measured values of the dislocation density indirectly confirm the absence of martensitic transformation in Hadfield steel during deformation, otherwise, judging by the literature, the dislocation density was significantly higher.

The density of microtwins during tensile deformation increases up to 4·10^−8^ m^−2^ with an increase in the degree of deformation at ε = 40%, while during compression (rolling) it reaches 5·10^−8^ m^−2^ at ε = 90% (Figure 6b). According to Figure 6b, both types of deformation are characterized by a decrease in the growth of microtwins with an increase in deformation. If at the initial stages of deformation (from 0.10 to 0.40) the density of microtwins increased in direct proportion to the degree of deformation, then at a deformation of 0.40 the slope of the curves becomes flatter. Figure 6b shows that for tensile-deformed samples, the density of microtwins begins to increase only at ε = 0.05, which is consistent with the presence of two stages of plastic flow development II_1_ and II_2_.

The moment of inclusion of twinning in the deformation coincides with an inflection on the flow curve leading to a decrease in the strain-hardening coefficient, i.e., twinning plays a significant role in the deformation of steel. In this regard, the contribution of microtwins (ε*_tw_*) and dislocations (ε*_disl_*) to the deformation was determined under the assumption of the additivity of their contribution:

(2) $$\varepsilon =\varepsilon_{tw}+\varepsilon_{disl},\;\varepsilon_{tw}=0.5\gamma$$

**Figure 9:**
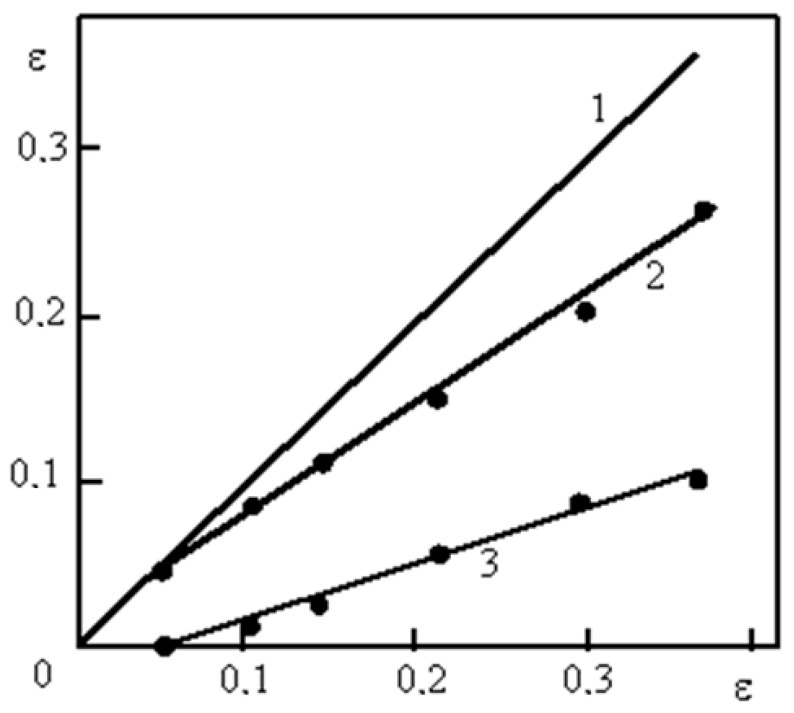
Contribution of dislocations (*2*) and microtwins (*3*) to the total plastic deformation ε (*1*).

In Equation (2) *γ* = *ρ_tw_*·*d*_0_, *d*_0_ is the width, and *ρ*_tw_ is the density of microtwins. The result obtained is shown in Figure 9.

**Figure 10:**
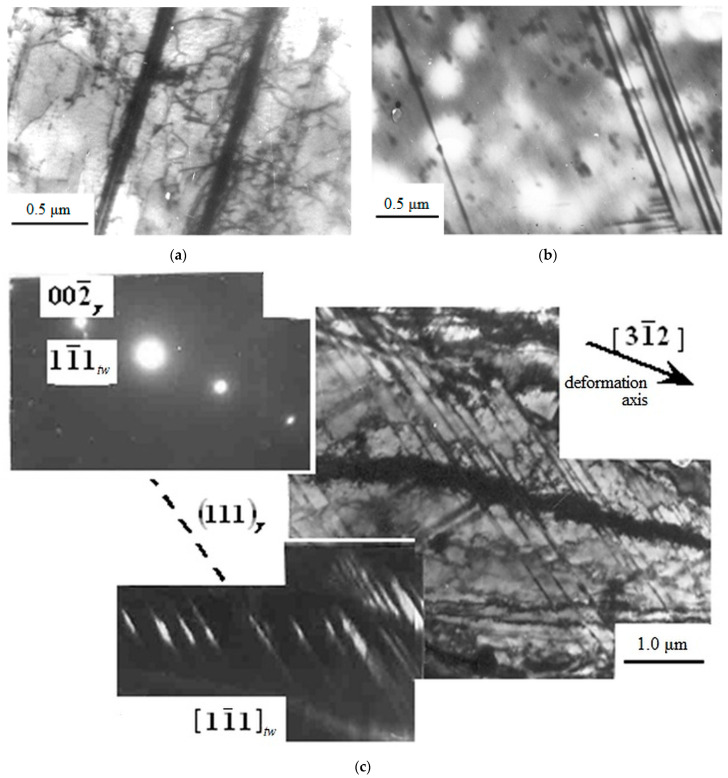
Microtwinning in Hadfield steel during tensile deformation with: (**a**) ε = 10%; (**b**) ε = 15%; (**c**) ε = 25%. The figure indicates the axis of deformation and the twinning plane trace (----), as well as the diffraction pattern with an indication of the twin [$1\bar{1}1$]_tw_ reflection, in which a dark-field image was taken.

Figure 9 shows that the contribution of twinning to deformation is quite significant (up to 1/3). Hence, it is not surprising that the appearance of twinning has such a strong influence on the value of the strain-hardening coefficient. The inclusion of twinning as an additional mode of plastic deformation should decrease the hardening rate. However, the result of the influence of twinning on the strain-hardening coefficient depends on the complications it introduces into the sliding process. Figure 10 shows TEM images of a sample deformed by tension with ε = 10%, 15%, and 25%. At the twin-matrix interface, dislocation tangles are observed (Figure 10a) indicating a strong deceleration of dislocations at the interface (ε = 10%). With an increase in the deformation degree to 15% and further to 25%, the number of twin boundaries free of dislocations increases (Figure 10b,c).

It is important to note that the TEM analysis of the structure of Hadfield steel did not reveal martensite anywhere, all the observed reflections of diffraction patterns are reflections of the γ-phase (Figure 7 and Figure 10c). The structure of the steel remains γ-austenitic even at degrees of rolling deformation of 40% (Figure 7) and higher, which is in full agreement with [11,20,22].

**Figure 11:**
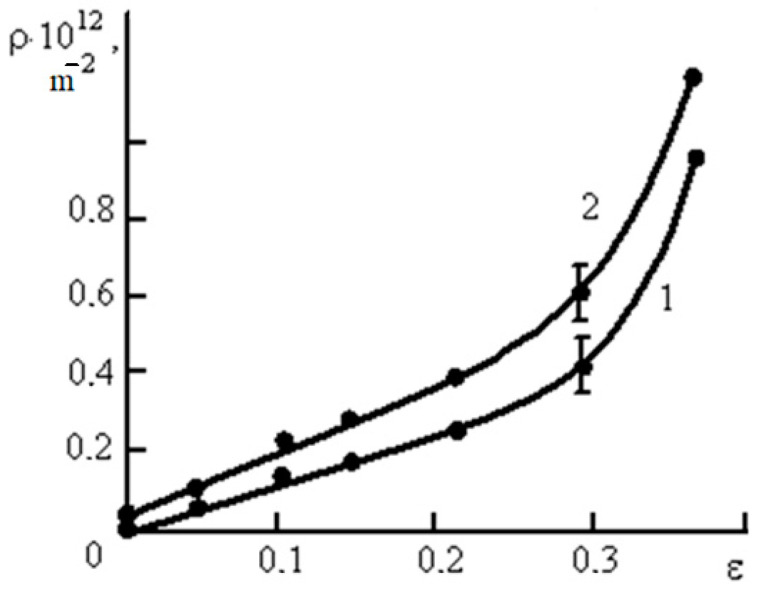
Dependence of the scalar dislocation density on the degree of plastic deformation of Hadfield steel for grains containing microtwins (curve 1) and for grains without microtwins (curve 2).

Since the density of dislocations in areas containing microtwins is lower, sliding through twins is easier than in the matrix, which is confirmed by the curves plotted based on the degree of plastic deformation of the scalar density of dislocations in grains containing and not containing microtwins (Figure 11).

The difficulty of sliding in the matrix can be due to two reasons: first, the resistance to the motion of sliding dislocations due to the presence of the microtwin boundary and, second, the magnitude of the orientation factor in the twin. Since the scalar dislocation density near the microtwins is different, then, apparently, the decisive factor will be the second reason, i.e., the value of the Schmid factor. An analysis of the dislocation structure (Figure 10c) showed that twinning develops along the (111)_γ_ plane. In this case, the condition of parallelism of the planes is fulfilled: (130)_γ_//(102)_tw_ and directions: [$\bar{3}15$]_γ_//[$\bar{4}\bar{2}2$]_tw_. The orientation of the tension axis in this area of the sample is [$3\bar{1}2$]_γ_. The TEM images (Figure 10c) were used to determine the change in the crystallographic indices of the deformation axis during twinning, and the Schmid factor (M) of sliding systems in the γ-matrix and twin was calculated using the Equation (3):

M = cos φ ∙ cos λ,
(3)

where *φ* is the angle between the deformation axis and the sliding plane; *λ* is the angle between the deformation axis and the sliding direction.

When twinning with respect to the pole (111)_γ_, the indices of the extension axis [$3\bar{1}2$]_γ_ change to [$\bar{1}50$]_tw_. The calculated Schmid factors for operating sliding systems in the matrix and twin are presented in Table 2.

Table 2 shows that the Schmid factors in the twin increase, i.e., mechanical microtwinning entails orientational softening and facilitates the sliding process.

**Figure 12:**
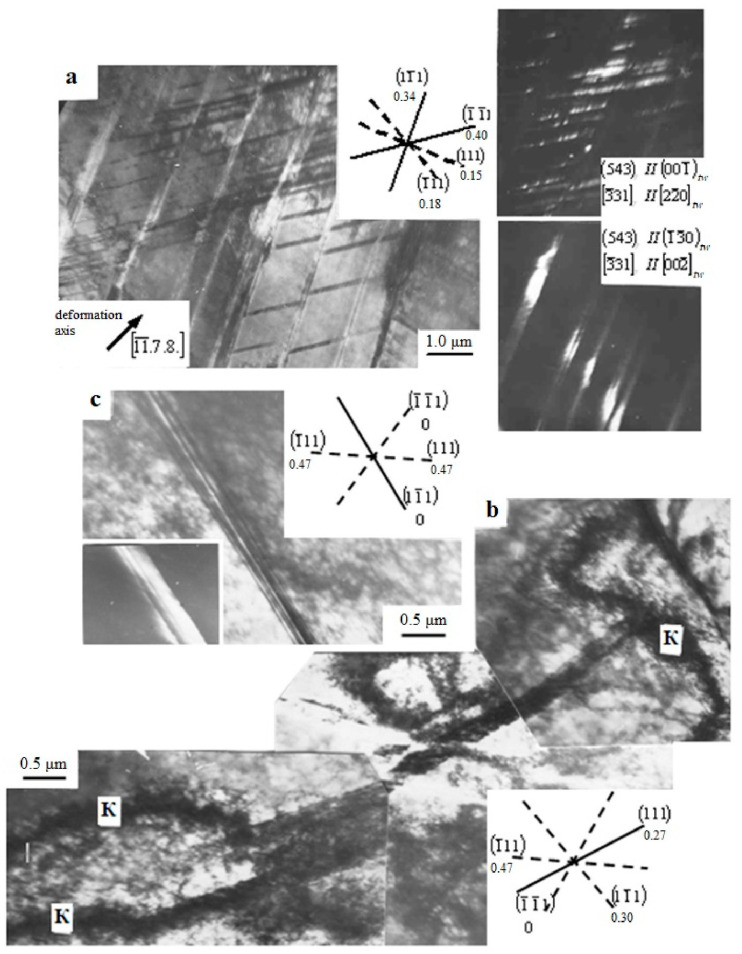
TEM micrographs of the microstructure of Hadfield steel deformed with ε = 25%: (**a**) samples after tension; (**b**,**c**) cold-rolled samples with an indication of the values of the Schmid factors, operating (solid line) and non-operating (dotted line) twinning systems, as well as extinction contour K.

To refine the geometry of twinning, microtwins were investigated in individual grains of steel samples deformed both by tension and rolling with ε = 25% (Figure 12).

As can be seen from Figure 12b,c, the formation of a cellular dislocation structure is observed in the rolled samples, which is noted as a characteristic stage in the evolution of the dislocation substructure of austenitic steels deformed by rolling in [20,38,39] at degrees of deformation exceeding 0.40. However, for samples deformed by tension, such a dislocation substructure at high degrees of deformation is not typical; most of the samples are destroyed when stretched by 0.40. There is also no experimental evidence of the glide plane softening, in which an ordered phase is sheared by some preceding dislocations, then the succeeding dislocations are easy to glide [19]. However, it is possible that the latter effect took place during tensile deformation at stages close to the failure of the samples, but its observation required the use of the in-situ TEM technique, as in [11].

The analysis of twinning geometry in specific grains showed that twinning develops mainly in systems with the highest Schmid factor (Figure 12a). However, twinning is observed with minimal (Figure 12b) and even zero (Figure 12c) Schmid factors. In these cases, twinning is apparently driven by internal stress fields due to the incompatibility of deformation of adjacent grains. This is evidenced by the presence of bending contours (Figure 12b). 

The results obtained indicate the important role of the slip plane orientation in newly formed microtwins and the Schmid factors for twinning. These results are also consistent with the assumption that the slip mechanism is facilitated by increasing the number of twinning systems and are in agreement with the theory of the twinning-induced plasticity effect, as described in [10,13,15,16,35,40]. The method proposed in this article for estimating the relationship between the stages of deformation and the parameters of the dislocation substructure of Hadfield steel by TEM methods is planned to be applied to the analysis of samples after impact fracture, supplementing it with the study of fractography using scanning electron microscopy. Future research aims to combine the obtained data on the relationship of substructure parameters with the mechanisms of various types of deformation of Fe-12.1wt.% Mn-1.2wt.% C steel to gain a fundamental understanding of high Mn austenitic steel deformation mechanisms.

## 4. Conclusions

The change in the course of the flow curve “σ − ε” (at ε = 5%) was accompanied by a sharp (1.5 times) decrease in the strain hardening coefficient and corresponded to the inclusion of the twinning mechanism in the deformation of steel.

At the deformation degree from 0 to 5%, the structure contains dislocation loops, the interweaving of elongated dislocations, single-layer stacking faults, and a cellular dislocation substructure. At deformation ε = 5%, the structure contains multilayer stacking faults and mechanical microtwins. At ε > 5%, there is an intense microtwinning in different systems, with no long dislocations and stacking faults in the material.

The most intense twinning with the formation of packets of twins develops in the range of deformation degrees of 5–20%, while the number of twins in the pack increases from 3–4 at ε = 10% to 6–8 at ε = 20%. Thus, there is a clear correlation between changes in the substructure of the Hadfield steel, the activation of new deformation (twinning) mechanisms, and the stages of plastic flow.

When mechanical twinning is included in deformation, a cellular dislocation substructure begins to develop intensively. The cell size decreases nonlinearly from 700 nm at ε = 5% to 150 nm at ε = 40%. 

It is found that the dependence of the scalar dislocation density on the degree of deformation up to 30% is parabolic; at higher degrees of deformation, the accumulation of dislocations becomes saturated.

The calculated Schmid factors for operating sliding systems in the matrix and twin showed that mechanical microtwinning entails orientation softening and facilitates the sliding process, while orientation hardening occurs at strains less than 5%. 

The results indicate the important role of the crystallographic texture in the mechanism of strain hardening of Hadfield steel and may be of interest to a wide range of researchers of the deformation processes of austenitic alloys. Knowledge of the features of work hardening of Hadfield steel can be used to improve the manufacture and processing of parts from it.

## Figures and Tables

**Table 1:** Elemental composition of samples of Hadfield steel of 110G13L grade (wt.%).

C	Mn	Cr	V	Si	P	S
1.19	12.1	-	-	0.39	0.012	0.015

**Table 2:** Schmid factors for existing sliding systems for the sample area in Figure 10c.

Sliding Systems	$[3\bar{1}2{]}_{\gamma }$	$[\bar{1}50{]}_{\mathrm{tw}}$
$\left(111\right)\{\begin{array}{c} \left[\bar{1}10\right]\\ \left[\bar{1}01\right]\\ \left[0\bar{1}1\right] \end{array}$	0.38 0.12 0.31	0.47 0.63 0.35

## Data Availability

The data presented in this study are available on request from the corresponding author.
